# Supplementary material for: Long-range and high-speed electronic spin-transport at a GaAs/AlGaAs semiconductor interface
Source: Sci Rep. 2016 Mar 16;6:22901. doi: 10.1038/srep22901 (PMC4793250; doi:10.1038/srep22901)
Supplement: Supplementary Information [file srep22901-s1.pdf]

Long-range and high-speed electronic spin-transport at a GaAs/AlGaAs semiconductor interface

Supplementary Information

L. Nádvorník,[1,2,*] P. Němec,[2] T. Janda,[2,1] K. Olejník,[1] V. Novák,[1] V. Skoromets,[3] H. Němec,[3] P. Kužel,[3] F. Trojánek,[2] T. Jungwirth,[1,4] and J. Wunderlich[1,5]

[1]*Institute of Physics ASCR, v.v.i., Cukrovarnická 10, 16253 Praha 6, Czech Republic*

[2]*Faculty of Mathematics and Physics, Charles University, Ke Karlovu 3, 12116 Praha 2, Czech Republic*

[3]*Institute of Physics ASCR, v.v.i., Na Slovance 2, 18221 Praha 8, Czech Republic*

[4]*School of Physics and Astronomy, University of Nottingham, Nottingham NG7 2RD, UK*

[5]*Hitachi Cambridge Laboratory, J. J. Thomson Avenue, CB3 0HE Cambridge, UK*

CONTENTS

Supplementary Figure S.1: Layer content and band diagram 3

Supplementary Figure S.2: Experimental time-resolved magneto-optical arrangements 4

Supplementary Figure S.3: Analysis of complicated Kerr dynamics 5

Supplementary Figure S.4: Evolution of spin life-time and amplitude 6

Supplementary Figure S.5: Discussion of the experimental estimate of μ_s 7

Supplementary Figure S.6: Sketch of Hall-bar patterning 8

Supplementary Figure S.7: Excess n and μ as function of temperature and illumination 9

Supplementary Figure S.8: Sketch of THz experimental arrangement 10

Supplementary Figure S.9: Fluence dependency of THz spectra 11

Supplementary Note 1. Electron-hole separation 12

Supplementary Note 2. Time-resolved magneto-optical setups 14

Supplementary Note 3. Analysis of magneto-optical dynamics 16

Supplementary Note 4. Robustness of magneto-optical signals 19

Supplementary Note 5. Experimental evaluation of D_s and μ_s 20

Supplementary Note 6. Methods for electrical experiments 22

Supplementary Note 7. Temperature and laser power dependencies 24

Supplementary Note 8. Setup and method for THz experiments 25

Supplementary Note 9. Additional THz results 27

References 27

Supplementary figures

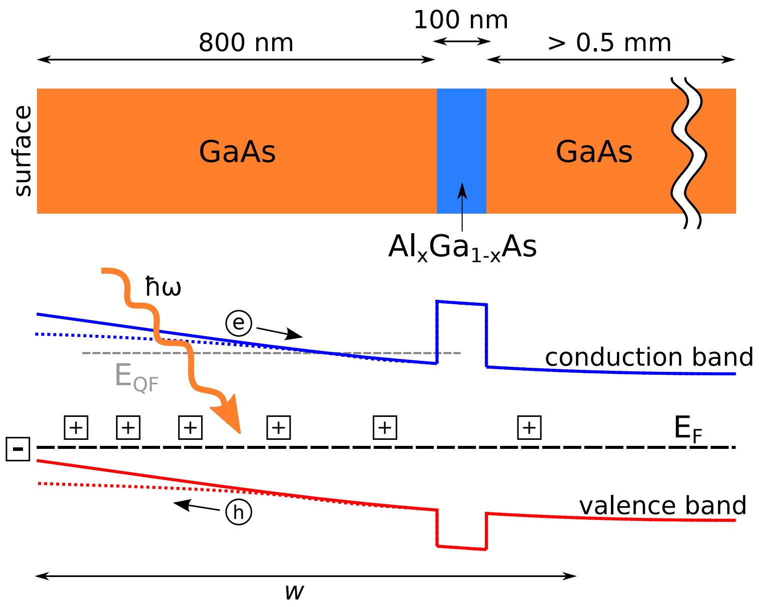

Supplementary Figure S.1. Sketch of the typical layer composition and thicknesses of used samples with the corresponding band diagram. It shows the band bending before the illumination (solid curves) due to the negatively charged surface states (square with the negative sign) and ionized unintentional impurity states in the bulk (squares with the positive signs); E_F is the Fermi level. After the illumination by the pump light ($\hbar\omega$) the electron-hole pairs are created (e, h letters in circles) and submitted to the built-in electric field, resulting in migration of photo-holes towards the surface and photo-electrons towards the barrier and the partial suppression of the band bending (dotted curves). The formation of the steady-state electron population near the interface is depicted by the quasi-Fermi level E_{QF}.

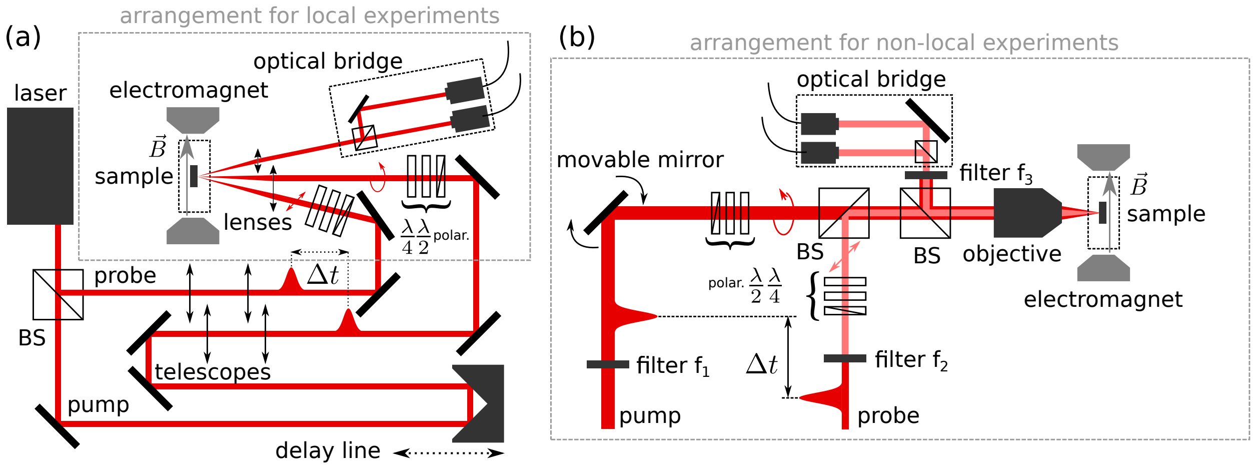

Supplementary Figure S.2. Experimental time-resolved magneto-optical arrangements. (a) Sketch of the whole pump and probe setup. The gray dashed rectangle depicts the arrangement for the local experiment with overlapped light spots. (b) A part of the setup arranged for the non-local experiments, allowing to spatially separate the pump beam relatively to the probe one. Beams are disjunctively spectrally filtered by filters f_1, f_2 and f_3.

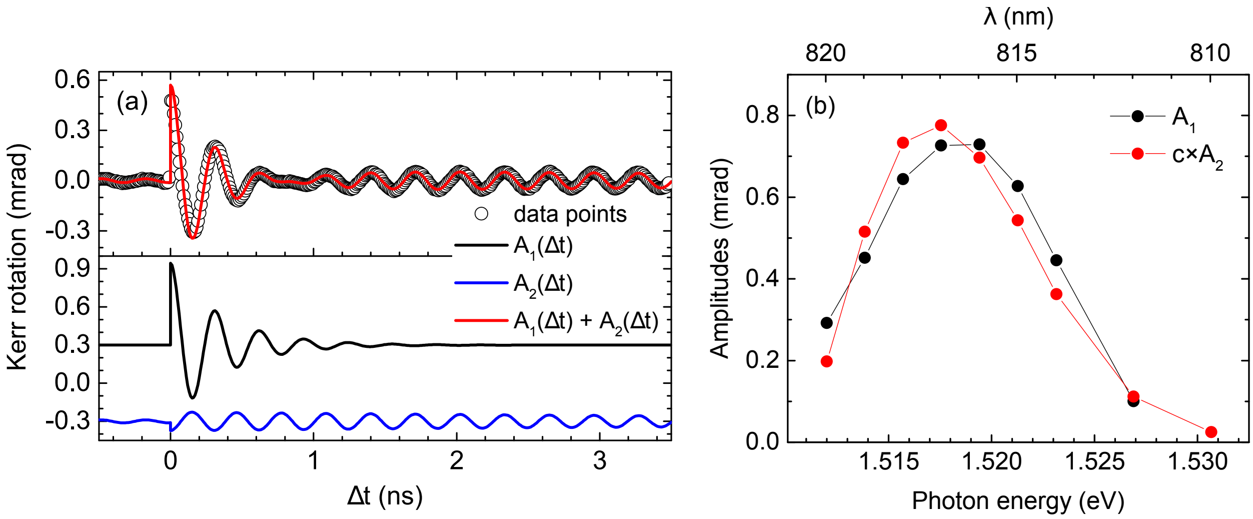

Supplementary Figure S.3. Analysis of complicated Kerr dynamics. Left panel: An example of the decomposition of the rather complicated signal (upper panel) to two components $A_1(\Delta t)$ and $A_2(\Delta t)$ (lower panel). The signal was measured in the structure (A) as defined in Fig. 4 in the main text. Right panel: The spectral dependence of the amplitudes A_1 and A_2, where the normalization factor $c = -9$ is chosen for clarity.

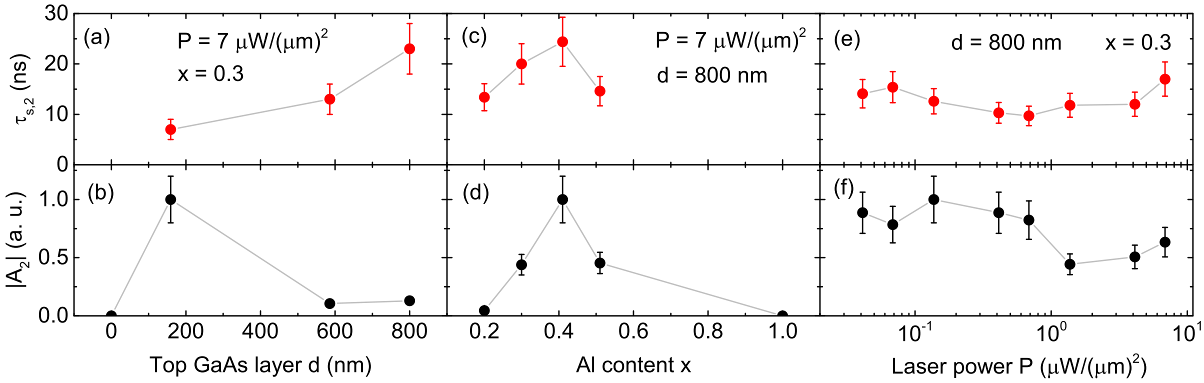

Supplementary Figure S.4. The evolutions of the spin life-time $\tau_{s,2}$ and the amplitude A_2 as functions of the thickness of the upper GaAs layer d (a, b), aluminium content x of the $\mathrm{Al}_x\mathrm{Ga}_{1-x}\mathrm{As}$ barrier (c, d) and laser illumination power P (e, f) for the conditions indicated by the text labels in the panels. See Fig. 2b in the main text for the sample structure. All data were measured in the local setup arrangement at $\lambda = 815$ nm, $T = 10$ K and $B = 500$ mT. The corresponding laser fluence is equal to $P/80$ MHz.

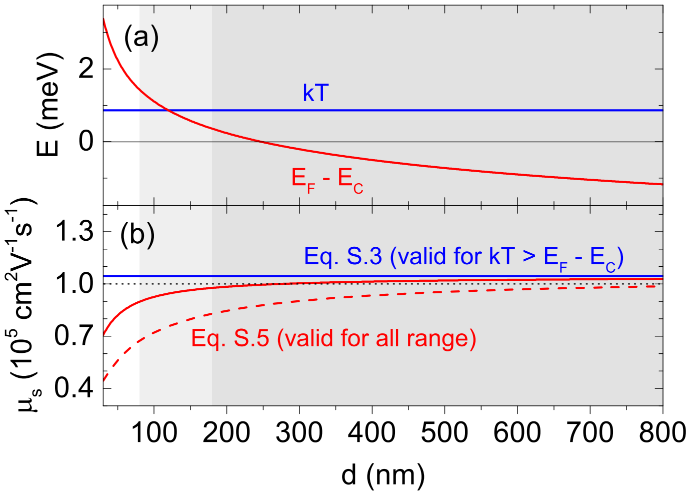

Supplementary Figure S.5. Discussion of the experimental estimate of μ_s. (a) Comparison of value of $E = kT$ (blue line) and $E_F - E_C$ (red curve, using Eq. S.3) with respect to the thickness d of the steady-state electron system. The darker, lighter gray and white area depict the non-degenerate ($kT > E_F - E_C$), transition ($kT \approx E_F - E_C$) and degenerate ($kT < E_F - E_C$) regime, respectively. (b) Dependence of μ_s on d using Eq. S.2 (blue line) and Eq. S.4 for the expected scattering on stationary phenomena (red solid curve) and for the scattering on moving phenomena (red dashed curve).

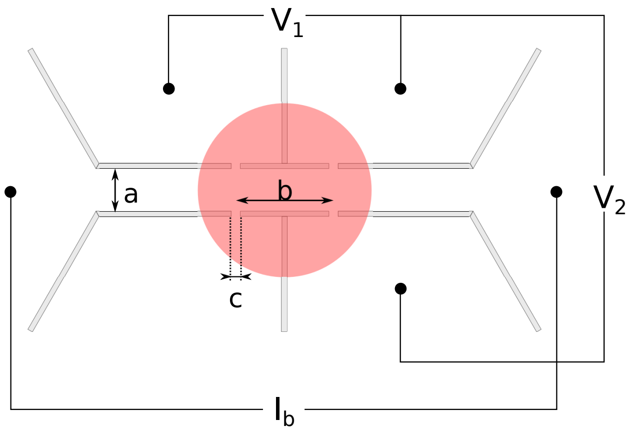

Supplementary Figure S.6. A sketch of Hall-bar patterning on the surfaces of samples, allowing to measure the 4-point longitudinal voltage drop V_1 and transversal Hall voltage V_2 when current biased to $I_b = 10$ μA. The red circle over two Hall crosses depicts the area illuminated by the fs- or cw-laser beams. The indicated dimensions are $a = 7$, $b = 14$ and $c = 2$ μm.

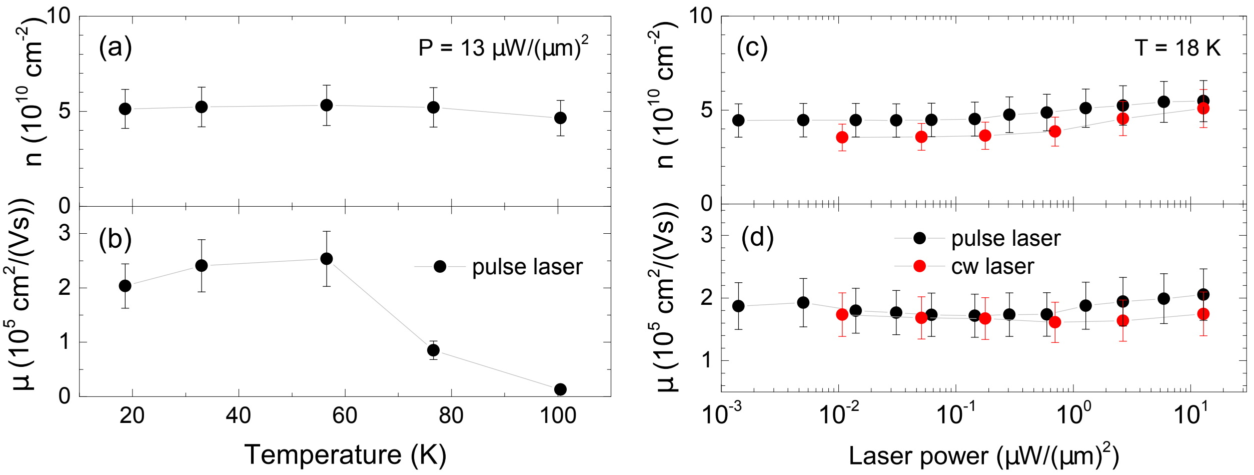

Supplementary Figure S.7. The excess electron density n and mobility μ as function of temperature and illumination powers for fs-laser (black points) and cw-laser (red points) excitation with $\lambda =$ 815 nm. The laser power $P = 13$ μW/μm^2 used in temperature measurements leads to the photocarrier concentration of 4×10^{13} cm^{-2}.

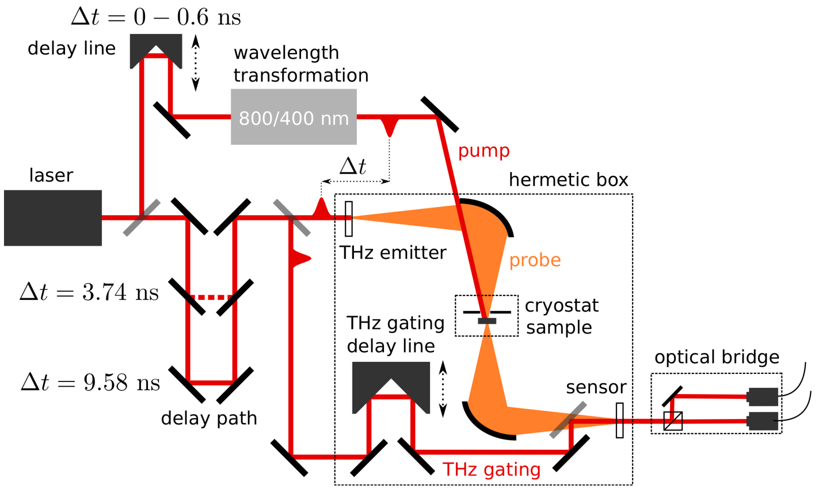

Supplementary Figure S.8. Sketch of the THz experimental arrangement. The red beams depict the near infrared laser light and the orange beam represents THz radiation.

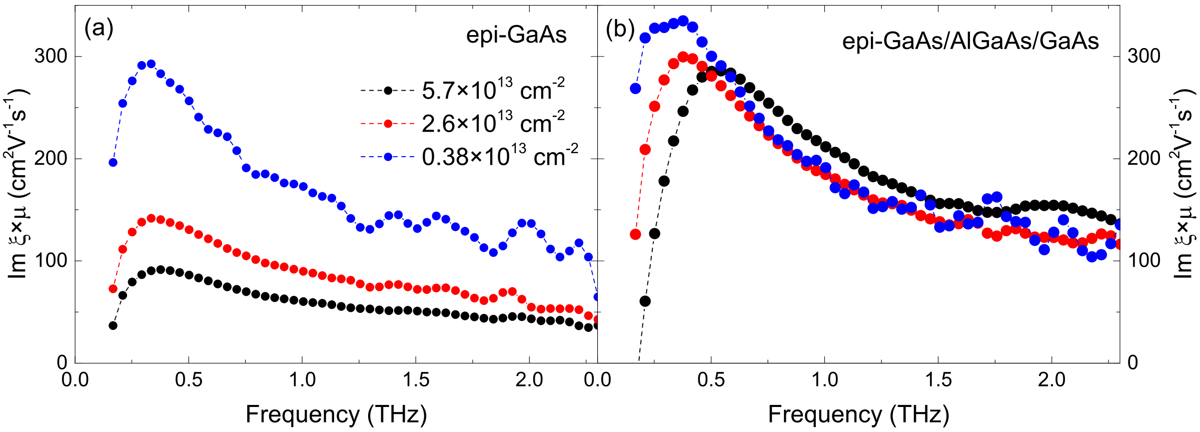

Supplementary Figure S.9. The imaginary parts of THz spectra measured on the reference epitaxial GaAs on GaAs substrate ((a), epi-GaAs) and on GaAs sample with AlGaAs barrier ((b), epi-GaAs/AlGaAs/GaAs) for various fluences generating the indicated photocarrier concentration per each pulse. The panel (a) represents a carrier-concentration-dependent decay which is usually observed. The absence of such a dependence in the panel (b) is a signature of affected electron-hole recombination by the presence of the built-in electric field. Data were measured at 18 K with wavelength $\lambda = 800$ nm and $\Delta t = 3.7$ ns.

Supplementary Note 1. ELECTRON-HOLE SEPARATION

The mechanism of electron-hole separation proposed in the main text is based on the presence of the built-in electric field generated by charged surface and bulk impurity states. The states on GaAs surface and their notable role in the structure growth and the device engineering have been reported for many decades[1,2]. The Fermi level pinning and the band bending are common manifestations of charged surface states and are usually taken into account when designing the doping and layer composition of GaAs-based structures. Our estimated values of the density of surface states $N_s \approx 10^{11} - 10^{12}$ cm^{-2} and of bulk impurity states (unintentionally introduced during the growth) $N_b \approx 10^{15}$ cm^{-3} are based on matching of numerical band calculations with electrical properties of grown structures and are in a reasonable agreement with the generally reported values[3,4]. As the Fermi level in our samples is surface-pinned near the valence band, the surface states are negatively charged by electrons provided by bulk impurity states that, thus, become positively charged (see Supplementary Fig. S.1). The thickness w of the space-charge region affected by the resulting band bending is estimated as $w \approx N_s/N_b = 1 - 10$ μm for considered values of N_s and N_b. We note that we use the orientation of the sample with its surface oriented upwards when we refer to the upper and lower layer or interface.

The steady-state electric field is present in dark. When the sample is illuminated, electron-hole pairs are created and the built-in electric field tends to separate them: holes are attracted towards the surface and electrons are forced in the opposite direction. The photo-carriers start to fill the charged surface states and the charged bulk impurity states and, thus, suppress the original electric field. If an undoped GaAs without any AlGaAs barrier is first considered, the photo-carriers can propagate freely to fill all the states and, therefore, the electric field is suppressed completely and the system does not exhibit any separation mechanism for the newly photo-generated carriers.

However, if the AlGaAs barrier is present in the depth $d = 800$ nm ($d < w$), there is a non-zero density of positively charged bulk impurity states that are located below the barrier. As the barrier insulates electrically the regions with negatively and positively charged impurity states (the upper and bottom GaAs layers), there is never a complete suppression of the built-in field possible, unlike in the sample without the barrier. We demonstrate the incomplete suppression in case of the illumination by two different wavelengths used in our experiments.

When the sample is illuminated by the near-infrared light ($\lambda \approx 800$ nm) with the penetration depth ≈ 1 μm, the electron-hole pairs photo-generated below the barrier are electrically insulated from the surface. In this region, the photo-electrons fill all the impurity states but the photo-holes cannot migrate through the barrier to fill the surface states (the second opposite surface is separated by a 0.5 mm thick substrate) and remain in this location. The photo-hole generated in the upper layer (left GaAs layer in Supplementary Fig. S.1) fill all the surface states but only a part of the generated photo-electrons are consumed to fill the bulk impurity states available in this layer. The remaining photo-electrons in the upper and photo-holes in the bottom GaAs layer represent the uncompensated electric field. Therefore, the remaining photo-electrons in the upper layer tend to localize in the potential minimum near the upper barrier interface (left interface in Supplementary Fig. S.1) and form a long-lived steady-state electron subsystem.

A similar scenario occurs when violet laser light is employed in the presented THz experiments ($\lambda = 400$ nm, penetration depth ≈ 20 nm). Due to the electric insulation of the upper layer by the barrier, the photo-electrons generated near the surface cannot cross the barrier to fill the positively charged bulk impurity states remaining below the barrier. The built-in field is not again completely suppressed and long-lived steady-state electron population near the upper barrier interface is created.

The presence of the electron-hole separation in the upper GaAs layer was demonstrated by the THz experiments using both illumination wavelengths (Fig. 3b in the main text). The electron-hole recombination times are dramatically affected by the separation and a specific fluence dependence of amplitudes of THz spectra is observed (see Fig. 3b,c in the main text and Supplementary Fig. S.9). The presence of the long-lived electron system 200 μs after the previous pump pulse is also reported in Fig. 3c (main text) and it is supported by the long spin life-time measured by the time-resolved pump and probe technique (i.e. Fig. 2a in the main text).

Supplementary Note 2. TIME-RESOLVED MAGNETO-OPTICAL SETUPS

The experiments are based on the pump and probe technique using the polar Kerr effect that allowed the local and non-local measurements by the relative motion of pump and probe laser beams. The Ti:Sapphire tunable pulse laser provided 150 fs long pulses with a repetition rate of 80 MHz (i.e. $\Delta t_0 = 12.5$ ns time separation between two subsequent pulses). The laser light was divided by a beam splitter (BS) to the pump and probe beams and time delayed (by Δt) with respect to each other by a precision delay line (see Supplementary Fig. S.2(a)). The intensity of the pump beam was modulated by a chopper wheel operating at frequency $f \approx 2$ kHz. After being resized by telescopes, the polarization of the pump and probe beams was set to circular (σ_+ and σ_-) and linear (vertical) states, respectively. In the arrangement for local experiments, both beams were focused by a converging lens (10D) through a window of the optical cryostat in order to form overlapped ≈ 25 μm wide spots on the sample surface. The angles of incidence of the pump and probe beams were $\sim 4°$ and $\sim 8°$, respectively. While the reflected pump light was blocked in order to suppress the scattered excitation, the reflected probe beam was collected by another converging lens and after passing through a half wave-plate it was analyzed in the optical bridge. Here, the light was divided by a polarizing BS and both resulting beams were detected by Si-based detectors and analysed by a pair of phase-sensitive amplifiers (lock-ins) locked to the chopper frequency f. The differential signal of the two detectors is directly related to the rotation of the polarization plane of the probe beam, known as the Kerr rotation. The sample was kept at temperature $T = 10$ K and in in-plane magnetic field of 500 mT, generated between coils of electromagnet. For the local experiments, the wavelength was set to $\lambda = 815$ nm and the light power of the pump and probe beam was 5 and 0.5 mW, respectively.

The arrangement for non-local experiments is sketched in Supplementary Fig. S.2(b). In order to efficiently block the excitation light at the entrance to the optical bridge, the pump and probe beams were filtered by a pair of disjunctive sharp bandpass filters f_1 and f_2, respectively. The pump and probe beams were circularly and linearly polarized, respectively, and merged by a non-polarizing BS. After the filtering, the pump and probe beams had intensities 2.5 and 0.5 mW and were centered near $\lambda = 824$ and 814 nm, respectively. A near infrared 20× objective (with numerical aperture $NA = 0.4$) was employed to focus beams to spots of ≈ 2 μm (full width at half maximum). The piezo-tilting mirror holder,

used in the pump path, allowed us to tilt the incoming angle and thus to move the pump spot with respect to the probe one. (We note that the additional time delay of the pump and probe beams due to the tilting of the angle of incidence is < 100 fs.) After being reflected, the light was again collected by the objective and led to the optical bridge. Here, another filter f_3 was used in order to suppress the pump light.

Supplementary Note 3. ANALYSIS OF MAGNETO-OPTICAL DYNAMICS

Kerr rotation dynamics in both the local and non-local arrangements (typical example is plotted in Supplementary Fig. S.3(a) upper panel) were obtained from the differential signal from two detectors in the optical bridge. The Kerr dynamics have a rather complicated structure. Here we need to consider two decay components: a short-lived (with amplitude A_1 and lifetime $\tau_{s,1}$), and a long-lived (with amplitude A_2 and lifetime $\tau_{s,2}$)[5]. Besides that, since the time delay $\Delta t_0 = 12.5$ ns between two subsequent pump pulses is shorter than the typical $\tau_{s,2}$, it implies that also the signal induced by the preceding pump pulse needs to be considered:

$$A(\Delta t) = A_1 \cos(\omega_1 \Delta t) \exp\left(-\frac{\Delta t}{\tau_{s,1}}\right) + A_2 \cos(\omega_2 \Delta t) \exp\left(-\frac{\Delta t}{\tau_{s,2}}\right) + \\ + A_2 \cos(\omega_2(\Delta t + \Delta t_0)) \exp\left(-\frac{(\Delta t + \Delta t_0)}{\tau_{s,2}}\right), \quad \text{(S.1)}$$

where Δt is the time delay between the pump and probe pulses and ω_i are the precession frequencies of both contributions. The presence of the non-zero signal for $\Delta t < 0$ allowed us to fit more precisely $\tau_{s,2}$ as the decay of the Kerr signal due to the long-lived component is rather slow in the experimentally accessible positive time delays. An example of the fitting and corresponding contributions of two terms are shown in Supplementary Fig. S.3(a). The typical magnitude of $\tau_{s,1}$ was of the order of hundreds of ps, the long-lived spin life-time reached $\tau_{s,2} \approx 7$ ns in this case and up to $\tau_{s,2} \approx 20$ ns in the structures studied in the main text. We note that in the main text τ_s is used to refer to $\tau_{s,2}$.

The long-lived component in Supplementary Fig. S.3(a) was attributed to the spin polarization of the free long-lived electron system located near the upper GaAs/AlGaAs interface, which was originally created by the electron-hole separation due to a build-in electric field. According to the observations in the THz experiment (see the main text), the free electrons have dramatically increased electron-hole recombination time $\tau_r^{excess} >> \tau_{s,2}$ (a non-zero contribution to the THz spectra was detected after the time comparable to the separation of subsequent pump pulses ≈ 200 μs) and, thus, form a steady-state excess electronic population. This population acts as a transient effective n-doping. The spin polarization is injected into the sample via spin orientation by the circularly polarized pump light. When new spin-polarized electrons and holes are generated in the effectively n-doped region, the holes loose their spin rapidly and recombine with whichever electron in the system in time τ_r,

which allows to retain the spin polarization injected in the system for times longer than τ_r. The measured $\tau_{s,2}$ is then unaffected by the photo-carrier life-time τ_r, similarly as reported in real n-doped bulk GaAs samples[6,7].

The short-lived component is related to the spin-polarized photo-created electron-hole pairs outside the region with the effective n-doping. The THz experiments report the presence of remaining electric field after the arrival of a pump pulse (we refer to the observation of changes in electron-hole recombination dynamics in Fig. 3b in the main text). This field can be present in the system already in the steady-state together with the long-lived electron subsystem and/or can be restored by disturbing the electron-subsystem by the pump pulse. The remaining (or restored) electric field is not strong enough to suppress completely the electron-hole recombination and results in $\tau_r < 1$ ns (that is still longer than in the reference GaAs sample, see Fig. 3b in the main text) which is consistent with the measured spin life-time of this component $\tau_{s,1} < 1$ ns (which is clearly limited by τ_r). However, there is an evidence of another possible contribution to the short-lived signal associated with the effectively n-doped region and with an exciton-trion spin injection into it.

As the laser wavelength is set on or even below the band edge of GaAs bandgap, the possible spin injection channel is also through exciton-trion generation. This mechanism is only effective if there is an excess electron population present in the system (i.e. in the form of a real or effective n-doping), as described in details in Refs. 8 and 9. When a spin polarized exciton is photo-created in the region of excess electronic subsystem, it can either recombine with mean time τ_r (and contribute to the short-lived spin component in Supplementary Fig. S.3(a)), or bind another free electron to form a negatively charged trion. As the wavefunctions of both electrons in the trion are overlapped, the electron must have an opposite spin due to the Pauli exclusion principle. This means that the trion formation pumps effectively a spin polarization to the electronic sea. As the hole spin is already unpolarized before the trion life-time (which is comparable to τ_r[8]), the trion recombination involves each of the oppositely spin polarized electrons with the same probability and, thus, it does not affect the spin polarization of the excess electronic sea. This mechanism is supported by the observation of different spectral dependences of the short-lived (possibly excitonic) contribution (amplitude A_1) and free electron contribution (amplitude A_2) in Supplementary Fig. S.3(b), which agree with the findings reported in Ref. 9. The maximum of A_2 is slightly shifted to lower energies due to the contribution of directly excited trions

that have lower formation energy.

Summarizing this discussion, the observed long spin life-time $\tau_{s,2}$ has been associated with the excess steady-state electronic subsystem which has life-time roughly $\tau_r^{excess} \approx 200$ μs and carries the spin polarization with spin life-time $\tau_{s,2} \approx 20$ ns injected via spin polarized photo-created carriers. The contributions with considerably shorted $\tau_{s,1}$ are related to the electron-hole pairs or excitons that do not transfer their spin to the excess electronic system and their spin life-time is thus limited by their recombination time $\tau_r < 1$ ns.

Supplementary Note 4. ROBUSTNESS OF MAGNETO-OPTICAL SIGNALS

The observed long-lived spin subsystem is not restricted to one specific sample only but it shows a remarkable robustness with respect to possible deviations in the design of the structure. The qualitatively same behaviour was reproducibly observed in a variety of samples with different composition or structure parameters and prepared in different molecular beam epitaxy (MBE) systems designated for fabrication of high mobility structures.

Supplementary Fig. S.4 shows the dependence of spin life-time $\tau_{s,2}$ and the amplitude A_2 of the long-lived signal in locally measured Kerr dynamics on the thickness of the upper GaAs layer (panel (a,b)), the $x-$composition of the $Al_xGa_{1-x}As$ barrier (c,d) and laser power density (e,f). Up to factor of $2-3$, $\tau_{s,2}$ does not seem to be dramatically dependent on the structure parameters and is mostly independent of the excitation power, however, the strength of the effect (A_2) does vary with the growth parameters by factor up to 10. From that we conclude that the boundary conditions and the geometrical factors are important only for the efficiency of the separation of electron-hole pairs and affect probably the density of the excess electron subsystem. Once the subsystem is created and in steady-state, the mechanism of the spin relaxation of its carriers, and thus $\tau_{s,2}$, is not much influenced by these parameters.

The very weak dependence of $\tau_{s,2}$ and A_2 on the excitation power is the signature of a saturation regime. This is in agreement with the evolution of excess carrier density n measured by electrical Hall experiment (shown in Supplementary Note 5), reporting the small variation of n between 5.5 and 4.5×10^{10} cm^{-2}. This value of steady-state n also supports the saturation regime if we consider that the number of photocreated carriers in last 200 nm of GaAs varies in Supplementary Fig. S.4(e,f) roughly between 4×10^{13} and 5×10^{10} cm^{-2}, which is still above steady-state n.

Supplementary Fig. S.4(b) also shows that the studied signal increases with decreasing thickness of the upper GaAs layer and suddenly disappears when all the upper layer is removed. This is an experimental signature that the excess electron system is located rather near the upper side of the $Al_xGa_{1-x}As$ barrier which is consistent with our model. The corresponding Kerr dynamics for $d = 0$ nm is plotted in Fig. 2a (green points) in the main text.

Supplementary Note 5. EXPERIMENTAL EVALUATION OF D_s AND μ_s

The experimentally measured D_s can be used to derive the spin mobility μ_s which is an important parameter describing the spin system in terms of drift-based experiments and applications. The usual way to determine μ_s uses Einstein's relation[10] that relates these two quantities

$$\mu_{(s)} = \frac{eD_{(s)}}{kT}, \tag{S.2}$$

where k is the Boltzman constant and T the temperature. Considering $D_s = 90$ cm^{2}s^{-1} and $T = 10$ K we get the estimate of $\mu_s = (1.0 \pm 0.1) \times 10^5$ cm^{2}V^{-1}s^{-1}. This result is in a very good agreement with the carrier mobilities $\mu \approx 1 \times 10^5$ cm^{2}V^{-1}s^{-1} determined by the THz experiment and within a factor of 2 with 2×10^5 cm^{2}V^{-1}s^{-1} obtained from the Hall experiments (see the main text or Supplementary Notes 6–9).

However, the relation in Eq. S.2 is not valid for a highly degenerate semiconductor, i. e. when $kT << E_F$, where E_F is the Fermi level. In order to determine E_F, we used the common expression for the concentration of carriers in the conduction band[10,11]

$$n_{3D} = \int_{E_c}^{\infty} DOS_{3D}(E) f_F(E)\, dE = C(kT)^{3/2} F_{1/2}(\eta_F), \tag{S.3}$$

where $n_{3D} = n/d$ is the 3D density, n the 2D density and d the thickness of the steady-state electronic subsystem, E_C the energy of the bottom edge of the conduction band, $DOS_{3D} = C\sqrt{E}$ the 3D density of states, $C = 1/(2\pi^2)(2m^*/\hbar^2)^{3/2}$, where m^* is the effective electron mass in GaAs, f_F is the Fermi-Dirac distribution at considered $T = 10$ K, $F_j(x) = \int_0^\infty \zeta^j/(1+e^{\zeta - x})d\zeta$ is the Fermi-Dirac integral, and $\eta_F = (E_F - E_C)/(kT)$ is the reduced Fermi energy.

As the Hall experiments provide only the 2D sheet density of carriers $n \approx 5 \times 10^{10}$ cm^{-2} (see Supplementary Note 6), we calculated E_F for various possible d and plotted it in Supplementary Fig. S.5(a). Accordingly, the relation in Eq. S.2 is valid for all d, except for the region of highly compressed system with $d < 70$ nm, where $kT < E_F$. Therefore, we used generalization of Einstein's relation proposed in Ref. 12, valid in all $E_F/(kT)$ ratios,

$$\mu_{(s)} = \frac{e}{kT} D_{(s)} \frac{\Gamma(b+5/2)}{\Gamma(b+3/2)} \frac{F_{b+1/2}(\eta_F)}{F_{b+3/2}(\eta_F)}, \tag{S.4}$$

where $\Gamma(x)$ is the Gamma function and the factor b depends on the type of scattering involved and has value of 3/2 for scattering by stationary phenomena or $-1/2$ for scattering

by moving phenomena. The corresponding curves are plotted in Supplementary Fig. S.5(b). Here, we expect that the source of scattering at $T = 10$ K is stationary (red solid line). It is clear that Eq. S.4 starts to differ more significantly from the Einstein's relation (Eq. S.2) only in case of highly condensed system with $d < 70$ nm where the mobility tends more significantly below $\mu_s \approx 1 \times 10^5$ cm^{2}V^{-1}s^{-1}. We emphasize that $\mu_s > 1 \times 10^5$ cm^{2}V^{-1}s^{-1} is consistent with both estimates of the electron μ measured by the Hall and THz experiments and the compressing electric field (and the related spin relaxation mechanism) is rather weak considering the high τ_s measured by the time-resolved MO experiments.

Supplementary Note 6. METHODS FOR ELECTRICAL EXPERIMENTS

The electrical measurements of the Hall effect and sheet conductivity under optical excitation were designed in order to get independently electrical transport parameters of the excess electron subsystem, especially its mobility μ and steady-state carrier density n. All studied samples were undoped and showed unmeasurable conductivity in dark at low temperatures. However, following the saturation character of the excess electronic subsystem (Supplementary Fig. S.4(f)), when samples were illuminated in the center area by the laser light employed for the time-resolved magneto-optical (MO) measurement, all the samples containing the AlGaAs barrier became conductive (the reference samples without the barrier exhibited no measurable conductivity under the same illumination). This overall photoconductivity disappeared immediately after blocking the laser light and appeared reproducibly with the illumination, disregarding the laser power. This behaviour can be understood as a local illumination by the residual scattered light in the cryostat which was, according to the saturation also reported here below, sufficiently intensive to create locally the long-lived excess electron subsystem over all the sample. Consistently, it was experimentally verified that the conductivity originates from the upper GaAs layer by etching insulating trenches with different depths. We note here that some of the samples exhibited an increase of A_2 and/or $\tau_{s,2}$ in the time-resolved MO measurements when a small electrical current was applied through the optically studied area. We explain this behaviour by a charge homogenization in the excess electron subsystem and by a suppression of charge domains. A small effect of a residual perpendicular voltage on the profile of the built-in electric field is also possible.

All samples that manifested the long spin life-time showed this local photoconductivity, and vice versa. So, we chose one representative sample with $d_{GaAs} = 800$ nm and $x = 0.3$ for the further electrical investigation. The sample surface was patterned with a Hall bar design (sketched in Supplementary Fig. S.6) allowing to measure the transversal Hall voltage V_2 and the 4-point longitudinal voltage V_1 when the main channel was biased to $I_b = 10$ μA. In order to achieve similar experimental conditions to the time-resolved MO measurements, the central detection part between two Hall crosses was illuminated by the same femtosecond pulsed laser with $\lambda = 815$ nm and with a variable intensity (depicted by the red circle in Supplementary Fig. S.6). As the electrical detection is a dc sensing technique, we repeated

the experiments also with a continuous wave (cw) laser with same the wavelength and intensity to check whether the pulsed regime plays a crucial role. The sample was placed in the optical cryostat and cooled down to 18 K and the out-of-plane magnetic field B was changed from 0 to 500 mT.

The simultaneously measured voltages $V_1(B)$ and $V_2(B)$ allow to determine the sheet conductivity $\sigma = bI_b/(aV_1(B=0))$, where a and b are the dimensions of the Hall bar (see Supplementary Fig. S.6), and the Hall coefficient $R_H = V_2(B)/(BI_b)$. We are not able to rule out whether the hole subsystem, created at the surface and filling the surface states, contributes to the overall conductivity. Therefore we take the holes into account and show here that even if they contribute, their influence is small. If we assume that the measured conductivity $\sigma = \sigma_n + \sigma_p$ is composed of the conductivities of the electron σ_n and hole σ_p subsystems and assuming the equal carrier densities $n = p$ in these subsystems, it follows for the overall conductivity

$$\sigma = \sigma_n(1 + \beta^{-1}) \tag{S.5}$$

and for the Hall coefficient

$$R_H = \frac{r_H}{en}\frac{(1-\beta^2)}{(1+\beta)^2} = \frac{r_H}{en}\alpha, \tag{S.6}$$

where $\beta = \mu_n/\mu_p$ is the ratio of electron and hole mobilities in their subsystems, reaching typically $\beta \approx 10$ in GaAs at low temperatures[13–15], and r_H is the Hall factor dependent on concentration of ionized impurities. For our samples we consider $r_H = 1.1 - 1.6$.[16] The mobility of the excess electron subsystem is then calculated from the measured values as follows

$$\mu_n = \frac{1}{r_H\alpha(1+\beta^{-1})}R_H\sigma. \tag{S.7}$$

The fraction in Eq.S.7 reaches values $0.8-1.2$ for considered interval of r_H, which is reflected in error setting in Supplementary Fig. S.7. The electron mobility at 18 K reached $\mu = (2.0 \pm 0.4) \times 10^5$ cm^{2}V^{-1}s^{-1}.

Supplementary Note 7. TEMPERATURE AND LASER POWER DEPENDENCIES

The measured values of μ and n as functions of temperature and illumination laser power are shown in Supplementary Fig. S.7(a,b) and (c,d), respectively. The evolution of n with temperature demonstrates that the creation of the excess electronic subsystem is efficient up to 80 K. The temperature dependence of μ manifests a weak maximum near 60 K and slightly decreases below this temperature. This is a typical signature of a presence of residual ionized impurities with concentration of the order of 10^9 cm^{-2} in the last 200 nm of GaAs,[13] which is more than one order of magnitude smaller value than n. The ionized impurities probably originate from the uncompensated residual background impurities from the growth, discussed in the main text regarding the electron-hole separation and in Supplementary Note 1.

The saturation manifested in the dependence on the laser power is in agreement with the similar independence of spin-related signal reported in Supplementary Fig. S.4 (e,f) and in Supplementary Note 4). Moreover, this is also consistent with the very long life-time of the excess electronic subsystem, $\tau_r^{excess} \approx 200$ μs, observed in the THz measurements and assumed in our model of electron-hole separation. Here, for the pulse repetition time $\Delta t_0 =$ 12.5 ns, each pulse at the lowest fluence generates one order of magnitude lower concentration of carriers compared to the steady-state n. The system can exhibit a saturation behaviour at this fluence only if $\tau_r^{excess} > 100$ ns. In addition, the same values of μ and n were measured for the fs- and cw-laser illumination, which indicates a minimal role of the duration of laser pulses.

Supplementary Note 8. SETUP AND METHOD FOR THZ EXPERIMENTS

The transient THz conductivity spectra were measured in a custom-made setup for time-resolved THz spectroscopy (see the sketch in Supplementary Fig. S.8 and Ref. 17) with a Ti:sapphire laser amplifier (Spitfire ACE, central wavelength 800 nm, 1 mJ pulse energy, 5 kHz repetition rate) as a laser source. One part of the laser beam was used for the THz pulse generation and its phase-sensitive detection by means of the optical rectification and electro-optic sampling in 1-mm-thick (110)-oriented ZnTe crystals, respectively (see the probe and THz gating beams in Supplementary Fig. S.8). The whole THz part of the setup was enclosed in a hermetic box allowing us to achieve primary vacuum to avoid absorption on water vapor. Another part of the laser beam was used for optical pumping of samples either at the fundamental wavelength (800 nm, 1.55 eV) or at its second harmonic (400 nm, 3.1 eV). The pump beam was defocused to generate photocarriers homogeneously across the sample attached to a 3 mm aperture (less than 25% of the pump beam power was transmitted through the empty aperture at the sample position). The angle between the optical pump and the THz probe beams was 10°. Short pump-probe delays (< 650 ps) were controlled by a standard delay line.[17] For long pump probe delays we built two auxiliary optical benches allowing us to set rapidly the delays of 3.74 ± 0.03 and 9.58 ± 0.03 ns by flipping two optical mirrors in and out of the beam.

The experiments were done on the same set of samples as used in the other presented magneto-optical and electrical experiments, i.e. with the GaAs/AlGaAs/GaAs structures and with the reference samples containing epitaxial GaAs on GaAs substrate without any AlGaAs barrier. At low temperatures the mobility of free carriers depends on their concentration. The penetration depth of the optical pulse at the fundamental wavelength is comparable to the thickness of the epitaxial GaAs film (d = 800 nm) deposited on top of AlGaAs barrier. It means that a part of conduction band carriers is generated in the GaAs substrate below AlGaAs barrier. These carriers can diffuse even deeper into the bulk of the sample. The THz photoconductivity signal then may be a superposition of several Drude terms originating at various depths and characterized by different momentum scattering times. Therefore, quantitative interpretation of such spectra may be difficult. For this reason, the THz spectra used for the experimental estimate of the mobility were measured with the pump at 400 nm (penetration depth is ≈ 20 nm); in these experiments no carriers can

be generated in the substrate.

The samples were placed in an optical cryostat (Oxford, Optistat) with a pair of sapphire input windows (transparent for both optical pump and THz probe pulses) and a pair of thin Mylar output windows (transparent for THz probe pulses). In the experiment an optical chopper was positioned in the path of the optical pump laser beam; this scheme ensures that we experimentally detect the photoinduced change ΔE of the THz wave form transmitted through the sample. The transient wave forms were measured at 18 K for both pump wavelengths; the photocarrier density and the time delay after excitation were varied.

Supplementary Note 9. ADDITIONAL THZ RESULTS

The imaginary part of the transient THz spectra measured on the reference sample without any barrier for various laser fluences at $\lambda = 800$ nm is plotted in Supplementary Fig. S.9(a). The decreasing amplitude of the spectra with the photo-generated carrier density is a signature of bimolecular electron-hole recombination which is usually reported in undoped GaAs.[18,19] However, the corresponding measurements on the sample containing the barrier, plotted in Supplementary Fig. S.9(b), exhibits a clear independence of the fluence for $\omega > 0.5$ THz. This observation, together with the different time decay of the signal at $\omega = 0.6$ THz reported in Fig. 3b in the main text, prove that the electron-hole recombination is affected by the spatial separation due to the remaining built-in electric field.

We note that the THz spectra for $\omega > 0.5$ THz are related to the standardly mobile carrier system in GaAs. The carrier mobility that corresponds to this part of spectra is estimated to $\mu \approx 1 \times 10^4$ cm^{2}V^{-1}s^{-1} by fitting the formula in Eq. 4 in the main text. Consistently with observations made in Supplementary Fig. S.7(b), the second high mobility contribution to the THz spectra [($\mu_l \approx 1.0 \pm 0.2) \times 10^5$ cm^{2}V^{-1}s^{-1}, see the main text] was observed at low temperatures only ($T < 50$ K).

---

* nadvl@fzu.cz

1 Sze, S. M. & Ng, K. K. *Physics of semiconductor devices* (Wiley-Interscience, Hoboken, New Jersey, 2007), 3rd ed edn.

2 Wieder, H. H. Problems and prospects of compound semiconductor field-effect transistors. *Journal of Vacuum Science & Technology* **17**, 1009–1018 (1980).

3 Yablonovitch, E., Skromme, B. J., Bhat, R., Harbison, J. P. & Gmitter, T. J. Band bending, Fermi level pinning, and surface fixed charge on chemically prepared GaAs surfaces. *Appl. Phys. Lett.* **54**, 555–557 (1989).

4 Pashley, M. D., Haberern, K. W., Feenstra, R. M. & Kirchner, P. D. Different Fermi-level pinning behavior on n- and p-type GaAs(001). *Phys. Rev. B* **48**, 4612–4615 (1993).

5 Sprinzl, D. *et al.* Influence of n -type doping on electron spin dephasing in CdTe. *Phys. Rev. B* **82**, 153201 (2010).

[6] Crooker, S. A. & Smith, D. L. Imaging spin flows in semiconductors subject to electric, magnetic, and strain fields. *Phys. Rev. Lett.* **94**, 236601 (2005).

[7] Dzhioev, R. *et al.* Manipulation of the Spin Memory of Electrons in n-GaAs. *Phys. Rev. Lett.* **88**, 256801 (2002).

[8] Chen, Z. *et al.* Electron spin polarization through interactions between excitons, trions, and the two-dimensional electron gas. *Phys. Rev. B* **75**, 115320 (2007).

[9] Gerlovin, I. *et al.* Electron-spin dephasing in GaAs/AlGaAs quantum wells with a gate-controlled electron density. *Phys. Rev. B* **75**, 115330 (2007).

[10] Ashcroft, N. W. & Mernim, N. D. *Solid State Physics* (Holt, Rinehart and Winston, New York, 1976).

[11] Aymerich-Humet, X., Serra-Mestres, F. & Millan, J. An analytical approximation for the Fermi-Dirac integral F32 (η). *Solid-State Electronics* **24**, 981–982 (1981).

[12] Marshak, A. H. & Assaf, D. A generalized Einstein relation for semiconductors. *Solid-State Electron.* **16**, 675–679 (1973).

[13] Stillman, G. E. & Wolfe, C. M. Electrical characterization of epitaxial layers. *Thin Solid Films* **31**, 69 (1976).

[14] Kim, M., Bose, S., Skromme, B., Lee, B. & Stillman, G. Hall effect analysis of high purity p-type gaas grown by metalorganic chemical vapor deposition. *Journal of Electronic Materials* **20**, 671–679 (1991).

[15] Madelung, O., Rössler, U. & Schulz, M. (eds.) *Group IV Elements, IV-IV and III-V Compounds. Part b - Electronic, Transport, Optical and Other Properties*, vol. b of *Landolt-Börnstein - Group III Condensed Matter* (Springer-Verlag, Berlin/Heidelberg, 2002).

[16] Benzaquen, M., Walsh, D. & Mazuruk, K. Hall factor of doped n-type GaAs and n-type InP. *Phys. Rev. B* **34**, 8947–8949 (1986).

[17] Fekete, L. *et al.* Ultrafast carrier dynamics in microcrystalline silicon probed by time-resolved terahertz spectroscopy. *Phys. Rev. B* **79**, 115306 (2009).

[18] Tanaka, S., Kuwata, T., Hokimoto, T., Kobayashi, H. & Saito, H. Picosecond Dynamics of Optical Gain Duo to Electron-Hole Plasma in GaAs under Near Band-Gap Excitation. *J. Phys. Soc. Jpn.* **52**, 677–685 (1983).

[19] Kaiser, W. *Ultrashort Laser Pulses and Applications* (Springer, Berlin, Germany, 1988).
